# Diet as a Risk Factor for Cognitive Decline in African Americans and Caucasians with a Parental History of Alzheimer's Disease: A Cross-Sectional Pilot Study Dietary Patterns

**DOI:** 10.14283/jpad.2018.44

**Published:** 2018-11-30

**Authors:** A.C. Nutaitis, S.D. Tharwani, M.C. Serra, F.C. Goldstein, L. Zhao, S.S. Sher, D.D. Verble, Whitney Wharton

**Affiliations:** 1 Emory University, Department of Neurology, Atlanta, GA, USA; 2 Atlanta VA Medical Center & Emory University Department of Medicine, Atlanta, GA, USA; 3 Emory University, Department of Biostatistics and Bioinformatics, Atlanta, GA, USA; 4 Emory University, Department of Internal Medicine, Atlanta, GA, USA

**Keywords:** Alzheimer's disease, Diet, African-American, Prevention, Nutrition, Race, Cognition, Vascular

## Abstract

**Background:**

African Americans (AA) are more likely to develop Alzheimer's disease (AD) than Caucasians (CC). Dietary modification may have the potential to reduce the risk of developing AD.

**Objective:**

The objective of this study is to investigate the relationship between Southern and Prudent diet patterns and cognitive performance in individuals at risk for developing AD.

**Design:**

Cross-sectional observational study.

**Participants:**

Sixty-six cognitively normal AA and CC individuals aged 46–77 years with a parental history of AD were enrolled.

**Measurements:**

Participants completed a Food Frequency questionnaire, cognitive function testing, which consisted of 8 neuropsychological tests, and cardiovascular risk factor assessments, including evaluation of microvascular and macrovascular function and ambulatory blood pressure monitoring.

**Results:**

Results revealed a relationship between the Southern diet and worse cognitive performance among AAs. AAs who consumed pies, mashed potatoes, tea, and sugar drinks showed worse cognitive performance (p<0.05) compared with CCs. In addition, gravy (p=0.06) and cooking oil/fat (p=0.06) showed negative trends with cognitive performance in AAs. In both CC and AA adults, greater adherence to a Prudent dietary pattern was associated with better cognitive outcomes. Cardiovascular results show that participants are overall healthy. AAs and CCs did not differ on any vascular measure including BP, arterial stiffness and endothelial function.

**Conclusion:**

Research shows that dietary factors can associate with cognitive outcomes. This preliminary crosssectional study suggests that foods characteristic of the Southern and Prudent diets may have differential effects on cognitive function in middle-aged individuals at high risk for AD. Results suggest that diet could be a non-pharmaceutical tool to reduce cognitive decline in racially diverse populations. It is possible that the increased prevalence of AD in AA could be partially reduced via diet modification.

## Abbreviations

AA: African Americans
AD: Alzheimer's disease
CCs: Caucasians

## Introduction

**O**ver five million people in the U.S. are living with Alzheimer's disease (AD), and in the next thirty years, the prevalence will increase to over sixteen million (1). Individuals at high risk of AD include African Americans (AAs), who have a 64% higher chance of developing AD than Caucasians (CCs) (2), and individuals with a parental history of AD, who are ten times more likely to become afflicted themselves (3). In the absence of a disease-modifying treatment, it is critical that we identify modifiable risk factors to promote cognitive health and reduce AD risk. Current preventative efforts focusing on lifestyle interventions including diet, exercise, and cognitive training (4, 5). Importantly, midlife (40-65 years of age) is when the neuropathological AD related changes begin and when the impact of vascular risk factors begin to have lasting effects. Thus, middle age is the optimal time to implement an AD focused lifestyle intervention.

Research suggests that adherence to a healthy diet confers cognitive benefits in older populations (6, 7, 8). Such diets include the Prudent, Dietary Approaches to Stop Hypertension (DASH) and Mediterranean diets, characterized by fruit, vegetables, legumes, fish and olive oil. While these studies are encouraging, few studies have examined the potential influence of diet on cognition in middle-aged, ethnically diverse populations, who are at high risk for AD.

In addition to genetic contributions, the increased prevalence of AD in AAs may be a result of modifiable risk factors including dietary intake (9, 10, 11, 12). In a study examining the association between the Mediterranean diet and cognitive decline, AA participants who had higher adherence with the Mediterranean diet had slower cognitive decline compared to participants with less Mediterranean diet adherence (13). Furthermore, current literature suggests that geographic and racial differences in cardiovascular disease risk are associated with the Southern dietary pattern (characterized by fried foods, fats, eggs, organ and processed meats and sugar-sweetened beverages) and thus it is possible that this Southern dietary pattern may contribute to cognitive decline (14). These findings stress the need for prospective studies addressing the relationships between diet and cognitive function in racially diverse populations in the U.S (15).

The goal of this study was to assess the relationship between dietary patterns, vascular function, and cognitive decline, in a middle aged, diverse cohort at high risk for AD due to a parental history of AD. We hypothesize that a higher intake of a Southern dietary pattern and lower intake of a Prudent (healthy) in dietary pattern increases the risk for vascular dysfunction and cognitive impairment, especially among AA, compared to CC, adults.

## Subjects and Methods

### Study Sample

Sixty-six subjects enrolled in an ongoing NIH/NIA funded study (ASCEND PI: Wharton) and with a parental history of AD took part in this cross-sectional pilot observational cohort study. Parental history was confirmed via autopsy or probable AD as defined by NINDS-ADRDA criteria and the Dementia Questionnaire (16). Subjects received vascular and cognitive assessments under the IRB approved protocol.

### Demographic Information

Age, gender, level of education, income, exercise, smoking status, and depression was acquired via a selfreported survey. Exercise was reported as mean days per week of cardiovascular exercise (17).

Dietary Pattern Assessment: Diet was assessed via the Jackson Heart Study's shortened version of the Lower Mississippi Delta Nutrition Intervention Research Initiative Food Frequency Questionnaire (FFQ) (18). The questionnaire consists of 160 items and takes 20 minutes to complete. Participants self-reported quantity and frequency of food and drink consumption on an online survey at home via a secure, individual web link. Subjects were given a $15.00 gift card for completing the survey.

Food items from the FFQ were classified into the Southern or Prudent diets in accordance Reasons for Geographic and Racial Differences in Stroke (REGARDS) study guidelines (14). Food items including fried foods, fats, eggs, organ and processed meats and sugarsweetened beverages were classified as characteristic of the Southern diet (14). Healthy foods including fruits, vegetables, whole grains, and fish were classified as Prudent diet related items (19).

### Cardiovascular Risk Factor Assessment

Vascular measures were selected based on prior research with vascular function in individuals at risk for AD (20, 21). Participants underwent a one-hour fasting assessment including microvascular vasodilatory function, using digital pulse amplitude tonometry (EndoPAT) and macrovascular vascular function (assessed by flow mediated vasodilation (FMD)). In addition, a blood pressure (BP) assessment was obtained via 24-hour ambulatory BP monitoring (Spacelabs Healthcare©). We examined 24-hour average systolic and diastolic blood pressure and nocturnal dipping patterns, all of which have been linked to cognition and AD (22).

### Neuropsychological testing

Cognitive function was evaluated by a one-hour battery of eight neuropsychological tests in domains reportedly affected in early AD and susceptible to the effects of hypertension (23). The tests included: Montreal Cognitive Assessment (MOCA), Benson visuospatial memory task, Buschke Delay Memory Test, Trails A and B, Digit Span Backwards, Mental Rotation Test (MRT), and Multilingual Naming Test (MINT). These tests targeted specific AD related cognitive domains including: working memory, executive function (Trail- Making Test B) (24, 25), language (MINT) (26), verbal memory (Buschke) (27), visuospatial ability (MRT) (28) and global cognition (MOCA) (29).

### Data Analysis

Researchers utilized IBM SPSS Statistics Version 22 to test for group differences between AAs and CCs in demographics, vascular risk factors, and cognitive performance. We conducted independent two-sample t-test for continuous variables and chi-square test for characteristic variables, controlling for age, gender and education. As there is not sufficient power to detect an interaction of diet and race, we examined the association between diet and cognition in each racial group separately. Correlations between cognitive performance and foods were assessed using Pearson's r partial correlations controlling for education and age on the cognitive tests in which we found racial differences at p=0.10. Because eight cognitive tests were included in the analyses, the threshold of significance level using a false discovery rate approximation was adjusted such that a threshold p-value of 0.03 was used.

## Results

Table 1 shows the demographic characteristics for 21 AAs and 45 CCs. Participants were middle aged (M=58.6 years), mostly female (67.6%), and highly educated (83.8% graduate or postgraduate education). While AAs and CCs did not differ on demographics including age, education, exercise, smoking status, or self-reported depression, significant racial differences were present for gender and income, such that a larger percent of AA females than CC females participated in the study, and AAs reported significantly less income compared to CCs. Participants were generally very healthy and AAs and CCs did not differ on any vascular measure including BP, arterial stiffness and endothelial function.

**Table 1:** Demographic Characteristics and Cardiovascular Data for African Americans and Caucasians. (AA= African American, CC=Caucasian)

**Variable**	**AA (N=21)**	**CC (N=45)**
Age (years)	57.8 ± 7.7	59.0 ± 5.8
Gender (% Female)	85.7% *	57.8% *
College graduate or higher level	85.7%	84.5%
Income**		
Income $39,000 or less	38.1%	11.1%
Income $40,000-$79,000	38.1%	24.4%
Income $80,000 or more	23.8%	64.4%
Mean days/week performing cardiovascular exercise	1.5	1.7
Ever Smoker	19.0%	26.7%
Current Smoker	4.8%	8.9%
Depression	28.6%	17.7%
Systolic (mmHg)	129.8 ± 12.3	125.5 ± 12.8
Diastolic (mmHg)	77.0 ± 5.4	77.6 ± 9.7
% Nocturnal Dipping	6.5 ± 6.9	7.3 ± 6.3
FMD 60 s.	6.1 ± 6.1	5.9 ± 4.2
FMD 90 s.	4.3 ± 6.9	4.5 ± 3.8
EndoPat RHI	2.4 ± 0.8	2.3 ± 0.8
EndoPat AIx	28.8 ± 19.2	22.1 ± 17.4


*P < 0.05; ** P < 0.01; RHI=reactive hyperemia index; AIx= augmentation index; FMD= flow mediated vasodilation

Table 2 shows cognitive test results by race. Results show that CCs significantly outperformed AAs on global cognition (MOCA), naming (MINT), and executive function (Trails B) tests (all p values <0.05). In addition, results revealed a trend for CCs to outperform AAs in verbal memory (Buschke Delay) (p= 0.073).

**Table 2:** Means and standard deviations on cognitive tests in African Americans and Caucasians. (AA=African American, CC=Caucasian)

	**AA (N=21)**	**CC (N=45)**
MOCA	25.39 ± 2.40 *	27.0 ± 2.33*
Benson Delay	11.89 ± 3.35	10.78 ± 2.78
Buschke Delay	5.37 ± 3.33 †	6.88 ± 2.80 †
MINT	29.53 ± 2.09 *	30.7 ± 1.98*
MRT	17.37 ± 3.29	17.8 ± 5.50
Trails B	120.32 ± 102.45*	81.9 ± 42.56*
Backwards Digit Span	4.11 ± 1.20	4.78 ± 1.75
Backwards Digit Span Accuracy	5.47 ± 1.87	6.63 ± 2.94


†P < 0.1; * P < 0.05

Table 3 shows Pearson's r partial correlations between foods and cognitive performance, by race. Five of six southern foods show moderate to strong correlations with cognitive tests in AAs. In AAs, pies, mashed potatoes, and sugar drinks were correlated with cognitive performance (all p values <0.01) and trends were found with tea (p=0.04), gravy (p=0.06) and cooking oil/fat (p=0.06), such that AAs performed worse on cognitive tests with consumption of these foods. Results show that AAs were more negatively impacted than CCs by foods characteristic of the Southern diet. Conversely, CCs who consumed mashed potatoes (p=0.01) and sugar drinks (p<0.10) performed better on cognitive assessments. Foods characteristic of the Prudent diet, such as whole grain breads (p=0.04), baked fish (p=0.03), and grape juice (p<0.01), were positively associated with cognitive performance in CCs. In addition, 100% orange juice (OJ) showed a trend (p<0.10) of better performance on cognitive assessment in CC. The most pronounced relationship was seen with 100% grape juice, such that AAs consuming 100% grape juice performed significantly better on the MINT (p<0.01). Results suggest a stronger relationship between the Prudent diet and cognitive performance in CCs vs. AAs.

**Table 3:** Pearson's r correlations between cognition and foods by race for individuals who completed Food Frequency Questionnaire. (AA= African American, CC=Caucasian; 1-6=Southern Diet, 7-10=Prudent Diet)

	**MOCA**	**Busche Delay**	**MINT**	**Trails B**
1. Pies				
AA	-0.904**	0.534	-0.295	-0.431
CC	-0.082	-0.144	0.224	0.192
2. Gravy				
AA	-0.056	-0.483	-0.791†	0.047
CC	0.085	-0.298	0.157	-0.111
3. Mash Potatoes				
AA	0.693	-0.662	0.417	0.889**
CC	0.100	-0.128	0.441**	-0.034
4. Added Fat/oil while cooking				
AA	0.096	-0.733†	0.112	0.551
CC	0.089	0.092	0.062	0.057
5. Tea				
AA	-0.772*	-0.045	-0.686	0.179
CC	0.189	0.129	0.128	-0.160
6. Sugar Drinks				
AA	0.529	-0.890**	0.419	0.664
CC	0.369*	0.307†	0.308†	-0.299
8. Baked Fish				
AA	-0.520	0.442	-0.514	-0.023
CC	-0.155	-0.075	0.388*	0.071
9. 100% Grape Juice				
AA	0.098	0.201	0.977**	0.145
CC	-0.057	-0.198	0.162	-0.264
10. 100% Orange Juice				
AA	0.264	0.239	0.054	0.192
CC	0.023	0.332†	0.032	-0.211


†P<0.1; * P<0.05; **P<0.01

## Discussion

To our knowledge, this is the first study to report a relationship between diet and cognitive performance in healthy, racially diverse middle-aged adults with a parental history of AD. CCs outperformed AAs on cognitive tests of global cognition, language, and executive function. Racial differences on cognitive tests could not be explained by age, education, vascular risk factors, exercise, smoking, or depression. However, our results suggest that these differences may be partially attributed to dietary patterns specific to the Southern and Prudent diets.

A positive relationship between cognition and the Prudent (healthy) diet and a negative relationship between cognition and the Southern (less healthy) diet was observed. Similarly, Shakersain et al. recently identified a relationship between lower adherence to a Prudent diet and greater rates of cognitive decline [6]. Further, Seetharaman et al. reported that elevated diabetes risk, which is higher in AAs than CCs, is related to poorer performance on perceptual speed, verbal ability, spatial ability, and overall cognition (30). Foods in our study characteristic of the Southern diet, such as pies, tea, and sugar drinks, were negatively associated with cognitive performance and thus it is possible that this may be a result of the higher glycemic index of these foods. Our results also align with studies showing that a diet high in gravy or butter is associated with poor cognition in older adults (31). Further, we show that racial differences in diet such that AAs reported stronger alliance with the Southern diet than CCs. This finding is not unique to our study, as previous studies show that AAs are less likely to adhere to the DASH diet compared with CCs (32, 33). Our study highlights the need for culturally sensitive dietary interventions to combat cognitive decline in high-risk populations.

Only one Prudent item (100% grape juice) was correlated to cognitive performance in AAs, in contrast to five Prudent items (whole grain breads, mashed potatoes, baked fish, 100% grape juice and 100% OJ). The Prudent diet is nutrient dense, containing numerous nutrients with anti-inflammatory and antioxidant properties, including fiber, poly-unsaturated fatty acids, vitamins, minerals, carotenoids, and polyphenols, among others (6). Therefore, it is possible that the negative effects of elevated inflammation and oxidative stress, which is more prevalent among AAs, on cognitive health may be dampened by the effects of the Prudent diet (34, 35). The association between beverages and cognitive performance should also be noted. Individuals may be more consistent with their beverage choices, (i.e. coffee or OJ), than food choices, and thus beverages may associate more strongly with cognitive function due to a higher intake.

The need for advancements in preventative and treatment strategies in high-risk groups, including AAs is great (36). Results showed racial differences in the relationship between diet and cognitive performance. It is possible that dietary intake may be contributing to early cognitive decline in AAs, or preservation of cognitive functioning in CCs. This finding is important, as the current literature suggests that even though latelife positive dietary patterns may result in notable health improvements (19, 37), mid-life is the optimal time to incorporate these changes, before the irreversible AD cascade begins (38). Thus diet modification may hold promise as a modifiable risk factor for AD.

Strengths of this study include a comprehensive battery of neuropsychology testing and vascular measures k]and a middle aged k]racially diverse cohort at high risk for AD. Also the FFQ is both racially and geographically sensitive (18). Limitations of this pilot project include the small sample size and the overall health of the cohort. It is possible that diet may have a more pronounced impact in individuals with preexisting health complications. Next the FFQ does not include information regarding longitudinal food choices k]and these data should be collected in future studies (39).

In summary, our results stress the need for further research investigating the potential of dietary intake as a non-pharmaceutical intervention in individuals at risk for AD. Because AAs have an increased incidence and prevalence of AD (2, 40), investigation of modifiable risk factors that target this high-risk group is essential. Specifically, nutritional education and dietary interventions designed to shift individuals, particularly AAs, from Southern diets to healthier, Prudent – like diets, may be a cost efficient way to preserve cognitive function in otherwise healthy individuals.

*Funding:* This project was funded by the National Institute of Health (NIH) and in part by the Scholarly Independent Research at Emory (SIRE) Research grant for undergraduate students. The NIH and SIRE had no role in study design, collection, analysis or interpretation of data; in the writing of the report; or in the decision to submit the article for publication.

*Acknowledgments:* All persons who have made substantial contributions to this manuscript are listed as authors. Their contributions are listed below: Alexandra C. Nutaitis, BS: Designed research, Conducted research, Analyzed data, Wrote paper, Had primary responsibility for final content . Sonum D. Tharwani: Conducted research, Wrote paper. Monica C. Serra, PhD: Provided essential reagents or materials, Analyzed data, Wrote paper. Felicia C. Goldstein, PhD: Designed research, Wrote paper. Liping Zhao, MSPH: Provided essential reagents or materials, Analyzed data, Wrote paper

Salman S. Sher, MD: Conducted research, Provided essential reagents or materials, Analyzed data, Wrote paper

Danielle D. Verble, MA: Conducted research, Wrote paper

Whitney Wharton, PhD: Designed research, Conducted research, Analyzed data, Wrote paper Had primary responsibility for final content

*Sources of Support:* NIH-NIA under grants: NIH-NIA 5 P50 AG025688, K01AG042498, and U01 AG016976. Independent funding for the present pilot study was obtained through Emory University's Scholarly Inquiry Research Grant for undergraduate students (PI: Nutaitis).

*Conflict of interest:* No author has a conflict of interest to report.
